# Structural and Dipole-Relaxation Processes in Epoxy–Multilayer Graphene Composites with Low Filler Content

**DOI:** 10.3390/polym13193360

**Published:** 2021-09-30

**Authors:** Borys M. Gorelov, Oleksandr V. Mischanchuk, Nadia V. Sigareva, Sergey V. Shulga, Alla M. Gorb, Oleksiy I. Polovina, Volodymyr O. Yukhymchuk

**Affiliations:** 1 Department of Composite Materials, Chuiko Institute of Surface Chemistry of the National Academy of Sciences of Ukraine, 03164 Kyiv, Ukraine; bgorel@ukr.net (B.M.G.); alexandr.mischanchuk@nas.gov.ua (O.V.M.); microft2@ukr.net (N.V.S.); sergey.v.shulga@gmail.com (S.V.S.); 2 Faculty of Physics, Taras Shevchenko National University of Kyiv, 01601 Kyiv, Ukraine; fantality@ukr.net; 3 Department of Optics and Spectroscopy of Semiconductor and Dielectric Materials, V. Lashkaryov Institute of Semiconductor Physics of the National Academy of Sciences of Ukraine, 03028 Kyiv, Ukraine; yukhym@isp.kiev.ua

**Keywords:** polymer nanocomposites, epoxy, graphene multilayered, thermal destruction, dielectric permittivity, positron annihilation

## Abstract

Multilayered graphene nanoplatelets (MLGs) were prepared from thermally expanded graphite flakes using an electrochemical technique. Morphological characterization of MLGs was performed using scanning electron microscopy (SEM), X-ray diffraction analysis (XRD), Raman spectroscopy (RS), and the Brunauer–Emmett–Teller (BET) method. DGEBA-epoxy-based nanocomposites filled with synthesized MLGs were studied using Static Mechanical Loading (SML), Thermal Desorption Mass Spectroscopy (TDMS), Broad-Band Dielectric Spectroscopy (BDS), and Positron Annihilation Lifetime Spectroscopy (PALS). The mass loading of the MLGs in the nanocomposites was varied between 0.0, 0.1, 0.2, 0.5, and 1% in the case of the SML study and 0.0, 1.0, 2, and 5% for the other measurements. Enhancements in the compression strength and the Young’s modulus were obtained at extremely low loadings (C≤ 0.01%). An essential increase in thermal stability and a decrease in destruction activation energy were observed at C≤ 5%. Both the dielectric permittivity ($\varepsilon_{1}$) and the dielectric loss factor ($\varepsilon_{2}$) increased with increasing C over the entire frequency region tested (4 Hz–8 MHz). Increased $\varepsilon_{2}$ is correlated with decreased free volume when increasing C. Physical mechanisms of MLG–epoxy interactions underlying the effects observed are discussed.

## 1. Introduction

Since the discovery of graphene, significant progress has been achieved in the synthesis, research, and practical application of nanographene structures. Thus, graphene, which possesses unique properties required for commercial applications, such as high heat conductivity and electron conductivity, mechanical strength, chemical stability, and optical transparency, may be the most promising material for advanced nanoelectronics and optoelectronics [1,2,3,4]. The prospects for widespread application of graphene and graphene-based composites as photodetectors [5], solar cells [6], super capacitors [7,8], optoelectronic devices [9,10], biosensors [11,12], SERS substrates [13,14], and nanoliquids [15,16] have been demonstrated.

It should be noted that the properties of graphene itself and its derivatives essentially depend on the synthesis technique used, the dimensions and number of the graphene’s atomic layers, and the concentration of vacancy, edge and other structural defects, which impact the device’s performance considerably. The basic properties of 2D graphene, multilayered graphene, and 3D graphene-based composites are essentially different; therefore, studying the physical and chemical properties of graphenic materials creates a firm basis of scientific knowledge for achieving controlled changes in the wide spectrum of parameters of nanostructured carbonic materials.

Along with graphene-based composites filled with single- or double-layer particles, the functional properties of materials with multilayered graphene nanoparticles (MLGs) and their prospective applications have been studied intensively. In multilayer particles, the weak interaction of neighboring monoatomic graphene layers leads to a weak three-dimensionalization of physical properties. Electron scattering increases, while both heat and electrical conductivities decrease. Some parameters of polymer composites filled with highly purified thermally expanded graphite can exceed those of graphene-based composites [17]; however, this circumstance does not restrict the scope of applications for MLG-filled polymer composites.

MLGs can possess basal surfaces of large size, whereas their lateral surface is of nano-sized thickness. The number of graphene layers can exceed one hundred. In MLG-filled polymer composites, the contacts between the neighboring particles significantly distort both the physical and chemical properties. Therefore, in order to prevent the aggregation of particles and their coming into contact within the host polymer matrix, it is most efficient to use particles with a basal plane size of no more than several tens of μm as a filler [4,18]. Due to the particles’ size and surface reactivity, their spatial distribution and orientation in the host polymer matrix contact between particles has an impact on the polymer’s molecular structure and, accordingly, on the mechanical, thermic, and dielectric properties of the composites; therefore, the experimental results obtained in different laboratories are ambiguous [19,20,21,22]. Nevertheless, in the range of fillings C > 1% the mechanical parameters (compressive and tensile strength) of polymer composites filled with different graphene types are characterized by close values, albeit far from the theoretically predicted values [23,24,25,26,27,28]. However, in the interval of mass concentration C ≤ 1% the loading effect of MLGs on mechanical parameters of polymer composites is less studied [23,29,30].

MLGs, along with high mechanical strength, possess a basic surface with high reactivity as well as a nanoscale lateral one, making them promising for applications as fillers in polymer composites to improve their thermal stability [18,19,26,27,31,32]. As a rule, the heat resistance of MLG-filled polymer composites increases with increasing C. Moreover, MLGs are capable of changing the mechanism underlying the thermal destruction of polymer macromolecular chains. In particular, the appearance of an additional, thermal-oxidative destruction and the transformation of the two-stage matrix decomposition process into a one-stage one after loading a polymer matrix with graphene particles was reported in [32].

The atomic composition of the thermal destruction products of epoxy composites at low MLG loadings has been determined previously using thermal desorption mass spectrometry. It was established that loading polymer with MLGs has no effect on the atomic composition of volatile destruction products, but it changes both the desorption intensity and the outputs of the products [33].

It should be noted that both the mechanical parameters and the thermal stability of polymer composites are dependent on the surface reactivity of the filling particles, their spatial ordering in a host polymer matrix, and peculiarities of the interphase interaction. Hence, a character of the interface interaction and the molecular structure are key factors determining the performance of various polymer composites. The influence of the interface interaction on the polymer molecular structure has been studied using dielectric spectroscopy in many works. The frequency and concentration dependences of the dielectric permittivity ($\varepsilon_{1}$) and the dielectric loss factor ($\varepsilon_{2}$) for various graphenic composites filled with single-layer [34], double-layered [1,35], and multilayered (more than three layers) [36,37,38] graphene particles and their mixtures [39,40,41,42] have been investigated. It has been discovered that at prethreshold concentrations of conductive and dielectrical nanoparticles, the polymer matrix may demonstrate frequency and concentration behavior of $\varepsilon_{1}$ inherent to both dielectrical and metallic systems [43,44,45,46,47]. The special behavior of dielectric permittivity has been attributed to the specific molecular structure within the interphase area of the nanocomposites studied. In particular, some models have been proposed to describe the interphase area structure. These include: multicore and dual layer models [48,49], a model involving the concept of quasiconductive layers or filaments among graphene particles in the polymer matrix [50], some models involving charge exchange among neighboring graphene particles, dielectric filler particles or atomic groups in polymer chains [44,47], or among separate segments of polymer chains (so-called micro-capacity model) [51,52,53,54], and also a cluster model or model of flip-flop transitions [55,56].

The wide diversity of proposed theoretical models for polymer matrix structure evidences the debatable status of the problem of establishing the interface interaction mechanisms and peculiarities of the influence of the interphase area on the matrix’s molecular structure and thus on the composite’s overall properties. Therefore, the aim of this work was to investigate the interphase interaction and its effect on the molecular structure of epoxy resin nanocomposites filled with nonoxidized MLGs and, as a consequence, to construct a polymer structure model capable of describing the behavior of nanocomposites under mechanical or thermal loading, under loading by either a low-intensity alternating electro-magnetic field or a positron beam.

Epoxy composites filled with nonoxidized multilayered graphene particles with lateral dimensions of no more than 5 × 5 μm and a thickness of about 50 nm were studied. In the composites, the mass C values were no more than 5% and did not exceed the threshold value. Such C values excluded the formation of conducting graphene clusters. The studies were carried out using several experimental methods, including Raman scattering, static mechanical loading, thermal desorption mass spectrometry, broadband dielectric spectroscopy, and lifetime positron spectroscopy.

## 2. Materials and Methods

### 2.1. Materials

The commercially available CHS-EPOXY 520 (Spolchemie, Ústi nad Labem, Czech Republic) DGEBA-epoxy resin, with epoxy group content 5.21–5.50 mol/kg, Epoxy Equivalent Weight 182–192 g/mol was used as the host resin. Polyethylene-polyamine (PEPA, Silkor Ltd., Brovary (Kyiv region), Ukraine) was used as a curing agent. The structural formulas of the epoxy resin and hardeners can be found elsewhere [57].

MLGs were obtained from thermal-expanded graphite flakes of Ukrainian production using the electrochemical technique described by Xia et al. [58].

**Figure 1:**
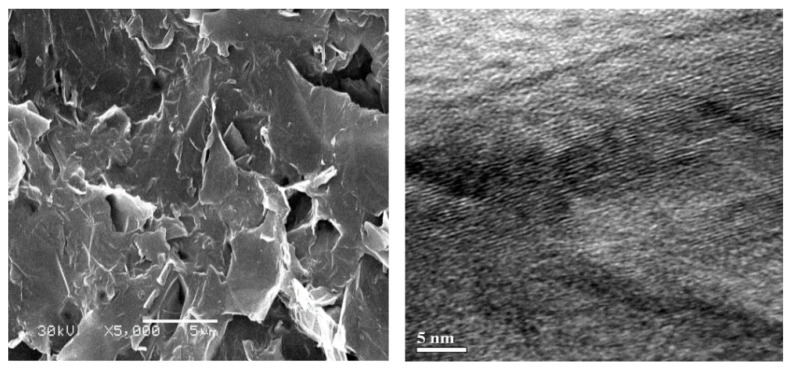
SEM images of basic and lateral surfaces of multilayered graphene particles.

The SEM study shows the so-prepared low-dimension particles to be multilayered graphene nanoplatelets with in-plane dimensions of about 5 × 5 μm and 50 nm in thickness. Both surfaces of graphene particles have a complicated morphology. There are edge defects, surface curvatures, waviness, and mesovoids on the basal surfaces. The lateral surface contains about a hundred graphene layers and is characterized by a disordered structure and roughness (Figure 1).

The specific surface value of $S_{f}\approx$ 740 m^2^/g was determined for neat MLGs by measuring the amount of physically adsorbed gaseous nitrogen from adsorption–desorption isotherms according to the standard Brunauer, Emmett, and Teller (BET) method [59].

**Figure 2:**
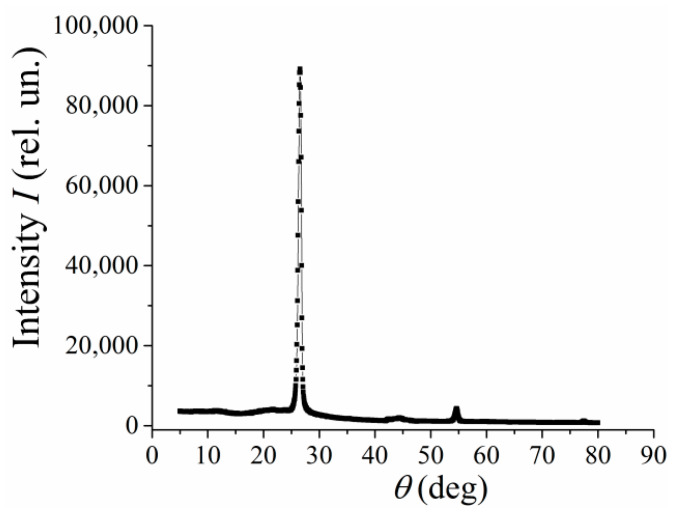
X-ray diffraction analysis of multilayered graphene particles.

The X-ray diffraction analysis shows that MLGs contain graphene sheets (Figure 2).

**Figure 3:**
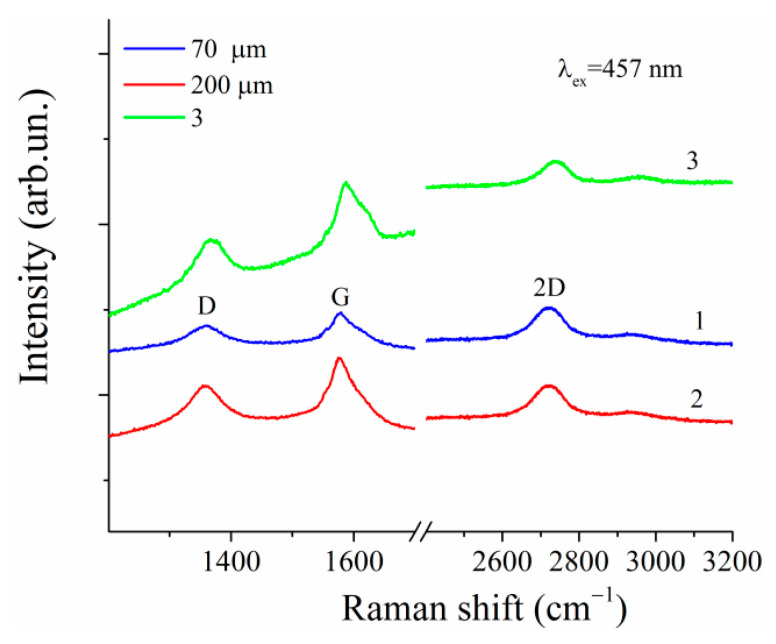
Raman spectra of oxidized multilayered graphene particles are recorded with spectrometer slit width 70 (**1**) and 200 μm (**2**). Spectrum of single-layer graphene (**3**) was taken from [2].

The first-order Raman spectra for both graphene and graphite contain the band G ~ 1582 cm^−1^, which belongs to the double degenerate mode $E_{2g}$ of the center of the Brillouin zone, and is due to tensile vibrations of $sp^{2}$ hybridized carbon bonds in benzene rings [2,60,61,62]. Additionally, defects in these carbon materials induce the D band at ~1350 cm^−1^. At the same time, analysis of the 2D band at ~2700 cm^−1^ in the Raman spectra is the most effective method to distinguish between graphene and graphite. The 2D band is due to scattering near the K-point of the Brillouin zone involving two phonons activated due to a double electronic resonance. The shape, position, and relative intensity of the 2D band in graphene differs significantly from the corresponding parameters of the 2D band in graphite. Analysis of both frequency position and shape of the 2D band in our Raman spectra (Figure 3, curves 1, 2), along with a comparison with the parameters of the corresponding 2D band in [2], evidence that graphene layers have really been formed.

Epoxy composites with nonoxidized graphene nanoplatelets were prepared by pouring a suspension of particles in ethanol into an uncured resin, to avoid the oxidation of graphene in air. The filler-mass-loading *C* in the nanocomposites was 1, 2, and 5% for thermophysical studies and 0.1, 0.2, 0.5 and 1% for mechanical measurements. As-prepared liquid composites were manually mixed until homogeneous suspensions were obtained, and their further polymerization occurred at room temperature during 72 h with following heat treatment at ~60 °C for 1.5 h. As a curing agent, 16% by weight of polyethylene polyamine was used.

### 2.2. Experimental Methods

#### 2.2.1. Raman Spectroscopy

Raman spectra were excited with 457 nm single-longitudinal-mode solid-state lasers (CNI Optoelectronics Tech. Co., Ltd., Changchun, China), with a power density on the samples of less than 105 W/cm^2^, which was low enough to preclude any thermal modification for samples studied. Dispersion of the spectra was performed using a spectrometer (DFS-52, LOMO, St. Petersburg, USSR) with the spectral resolution of 2 cm^−1^. The TE-cooled (−60 °C) CCD detector (Andor iDus 420, Belfast, UK) was used to detect the spectra. The spectra were measured in so-called “backscattering” geometry.

#### 2.2.2. Mechanical Static Loadings

Mechanical parameters, namely the compressive strength σ and the elastic (Young’s) modulus E were measured using the materials testing machine Z0.5 TS (ZwickRoell GmbH & Co., KG, Ulm, Germany) operating in compression mode. Samples for mechanical testing had a cylindrical shape with diameter and height about 10 mm. The values of the σ and E parameters were determined as arithmetic mean values over data obtained for five samples.

#### 2.2.3. Thermal Desorption Mass Spectroscopy

An influence of MLGs filler on the composite’s thermal stability has been determined using a programmable thermal desorption technique combined with a mass spectroscopic detection via the single-pole mass-analyzer MX 7304A (SELMI, Sumy, Ukraine). Mass spectrometric analysis of positive charged atomic products was performed in the m/z range 10–200 (m is the mass, z is charge of the fragment emanated), within a temperature range of 25–800 °C, at a pressure 0.1 Pa, and the heating rate 8 °C/min. The measurement details can be found elsewhere [63].

#### 2.2.4. Broad-Band Dielectric Spectroscopy

Dielectric properties of the neat resin and its composites filled with MLGs were obtained by measuring the complex relative dielectric permittivity $\varepsilon^{*}=\varepsilon_{1}+j\varepsilon_{2}$ using the computer-controlled impedance meter Hioki IM3536 (Hioki E.E.Corporation, Ueda, Nagano, Japan) General Purpose LCR Meter. The samples under study were prepared as circular-shaped plates having basal surface area of about (7–8) × $10^{-5}$ m^2^ and a thickness of about (1–2) × $10^{-3}$ m. Measurements of both the dielectric permittivity ε_1_ and the dielectric loss factor $\varepsilon_{2}$ were carried out within the temperature range 77–350 K at fixed frequencies, ranged from 4 Hz to 8.0 MHz. The temperature was changed at a rate of 0.5 K/min and was controlled to an accuracy of ±0.5 K. Details of sample preparation and measurement technique can be found in [64,65].

#### 2.2.5. Positron Life-Time Spectroscopy

The positron annihilation spectra for investigated materials were obtained using the “Ortec” (Ortec, Advanced Measurement Technology, Oak Ridge, TN, USA) positron lifetime system. “Scionix” modules equipped with two XP 2020Q photomultiplier tubes optically coupled with cylindrical BaF_2_ scintillators (∅ 25.4 mm, 10 mm) operated as the detectors of two different γ-quanta attributed to start (1274.6 keV) and stop (511.0 keV) of a positron lifetime. A 0.1 MBq ^22^Na positron source sealed by kapton foils (12.5 μm in thickness) was placed between two plates of the same sample. The time resolution of the device was determined to be 230 ps from the prompt curve of a ^60^Co source, at the temperature T = 18 °C. About a million annihilation events were collected to evaluate positron lifetime components $\tau_{1}$, $\tau_{2}$, $\tau_{3}$ and intensities $I_{1}$, $I_{2}$, $I_{3}$ from row positron annihilation lifetime spectra fitted by three components. The mathematical treatment was performed by using the LT 9.0 program [66].

## 3. Results and Discussion

### 3.1. Mechanical Loading Data

**Figure 4:**
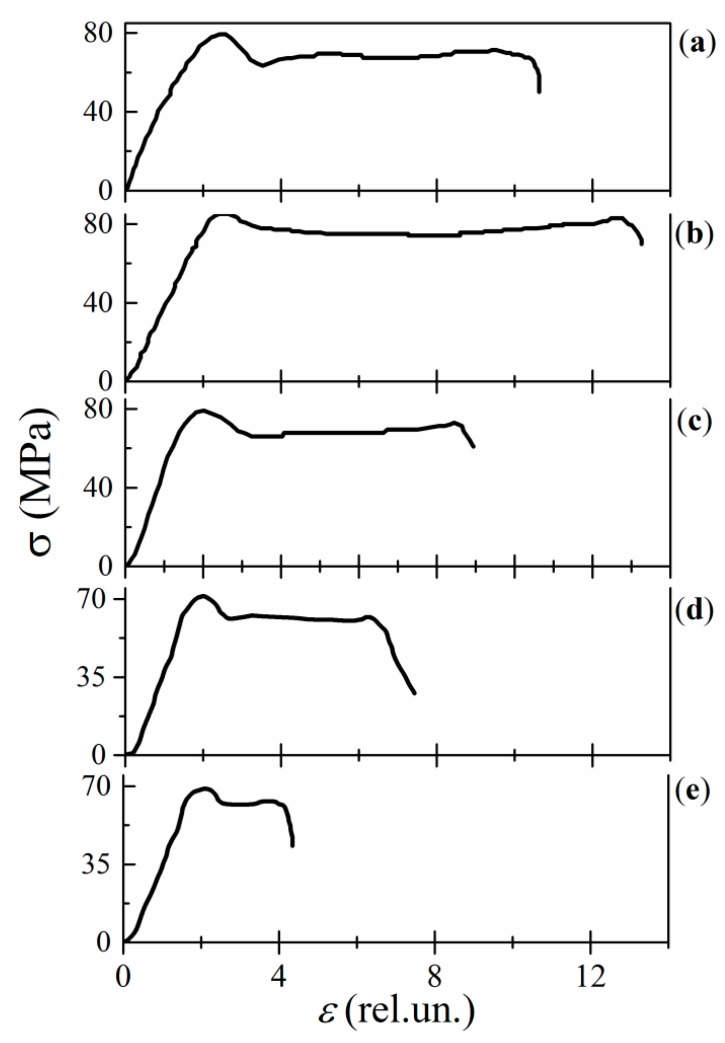
Stress–strain curves for the neat resin (**a**) and MLG composites of 0.01% (**b**), 0.1% (**c**), 0.5% (**d**), and 1% (**e**) MLGs.

The static compression-loading stress (σ)–strain (ε) curves for the neat epoxy resin and its MLG composites are shown in Figure 4. The curves demonstrate two loading intervals that are characterized by different physical mechanisms of deformation. In the deformation range of ε< 3, where strain increases linearly with ε, the deformation is elastic. The conformational plastic deformation occurs at ε> 2.5, when σ(ε) dependence remains practically unchanged, but irreversible configuration alterations take place in the polymer macromolecular structure. The configuration alterations are unavoidably accompanied by relevant alteration in the free volume of the composites.

**Figure 5:**
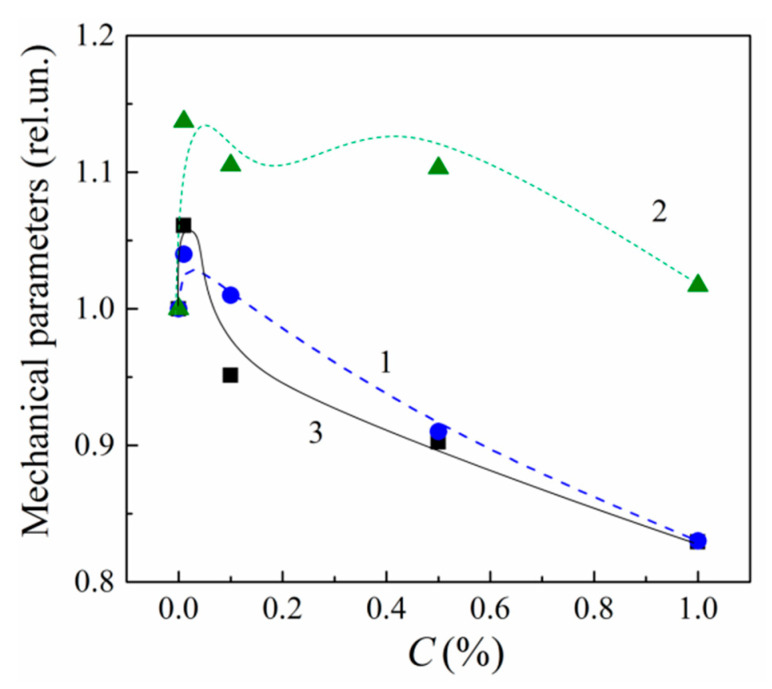
Concentration dependences of compressive strength ($\sigma_{0}$, 1), Young’s modulus (E, 2) and the conformational deformation (Δ*ε*, 3) for MLG–epoxy composites.

The concentration dependences of the compressive strength ($\sigma_{0}$) and the Young’s modulus (E) for MLG composites exhibit a similar nonmonotonous behavior with increasing C. Namely, both $\sigma_{0}$ and E values increase in the extremely narrow loading interval of C ≤ 0.01%, whereas they both decrease with C within the interval 0.01 <C≤ 1% (Figure 5).

The conformational deformation is inherent to the neat epoxy (Figure 4a). Its quantitative measure is $\Delta \varepsilon =\varepsilon_{0}-\varepsilon_{e}$, where $\varepsilon_{0}$ and $\varepsilon_{e}$ correspond the onset and ending of the plastic deformation in σ(ε)–curves. In the MLG composites, Δ*ε* varies nonmonotonously with increasing C. At C≤ 0.01%, both Δε and correspondent free volume (f.v.), where the conformational deformation takes place, increase (Figure 4b). However, in the concentration interval of 0.01 <C≤ 1%, Δε(C) and f.v. decrease gradually with increasing C (Figure 4c–e). Thus, the volume of conformational deformations reveals its nonmonotonic concentration dependence in the region C≤ 1%.

The behavior of the f.v. versus C is correlated with one of the parameters $\sigma_{0}$ and E (Figure 5). Loading with graphene particles changes the polymeric structure chains, the free volume of the composites, and their mechanical parameters in a similar way. Increasing f.v. at C≤ 0.01% leads to growth of $\sigma_{0}$ and E. In addition, vice versa, decreasing f.v. at C> 0.01% is accompanied by decrements in both $\sigma_{0}$ and E.

It should be noted that the alteration of the macromolecular structure and the corresponding variations in f.v., $\sigma_{0}$ and E occurring at C≤ 0.01% can be attributed to the fastening of unbound moieties of polymer chains with graphene’s active surface sites (ASSs). The fastening process decreases the faultiness of both epoxy and crosslinks. On the other hand, a frame of MLGs restricts the chain’s mobility. Numerical estimations show that there are 42 $\times 10^{3}$ MLG particles per 1 ${\mathrm{mm}}^{3}$ of 0.01% nanocomposite (provided that the particles are uniformly distributed over the bulk). Thus, these two effects lead to a more perfect and elastic molecular structure which is characterized by increased free volume and enhanced mechanical parameters at C≤ 0.01%.

In the concentration interval of 0.01 <C≤ 1%, the disordering of the macromolecular structure is increased due to the nonuniform spatial distribution of sites and the formation of interphase layers around MLGs. The free volume and mechanical parameters are decreased.

### 3.2. T. hermal Destruction Data

#### 3.2.1. Destruction’s Volatile Products

**Figure 6:**
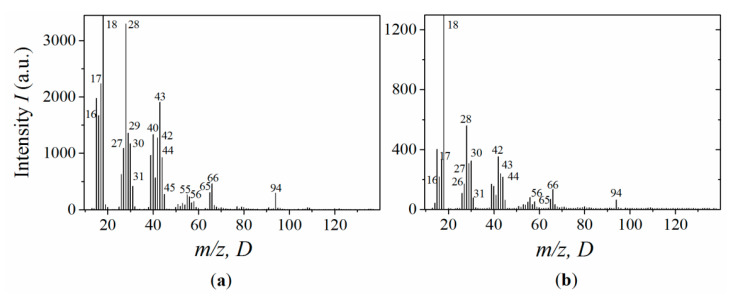
Mass spectra of the neat epoxy (**a**) and MLG composites of 1% (**b**). MLGs at temperatures corresponding to the maximum desorption intensity.

Figure 6 shows the mass spectra of atomic fragments of thermal destruction of the neat resin and its MLG composite filled with 1% MLGs. The mass spectra were obtained at temperatures corresponding to the maximal intensity of the lines. It can be seen that the rather strong lines correspond to the following m/z moieties: 14–18, 28–31, 40–45, 56–59, 65–67, 94. The majority of lines observed in the spectra can easily be identified by looking at the epoxy’s structural formula [57]. Among these lines, hydroxyl- and oxygen-containing fragments of m/z = 17 (OH), 28 (CO), 43 (CH_2_COH), and 94 (C_6_H_5_OH) originate from epoxy chains.

The embedding of MLGs into epoxy does not vary the total number of lines. However, the interaction of polymer atoms with the graphene’s ASSs significantly affects the thermal decomposition intensity of the epoxy matrix and its thermal stability. The loading of epoxy with MLGs enhances the thermal stability of the composites. The latter is clearly manifested upon C= 1%, when the line’s intensity decreases by ~3–7 times (Figure 6).

**Figure 7:**
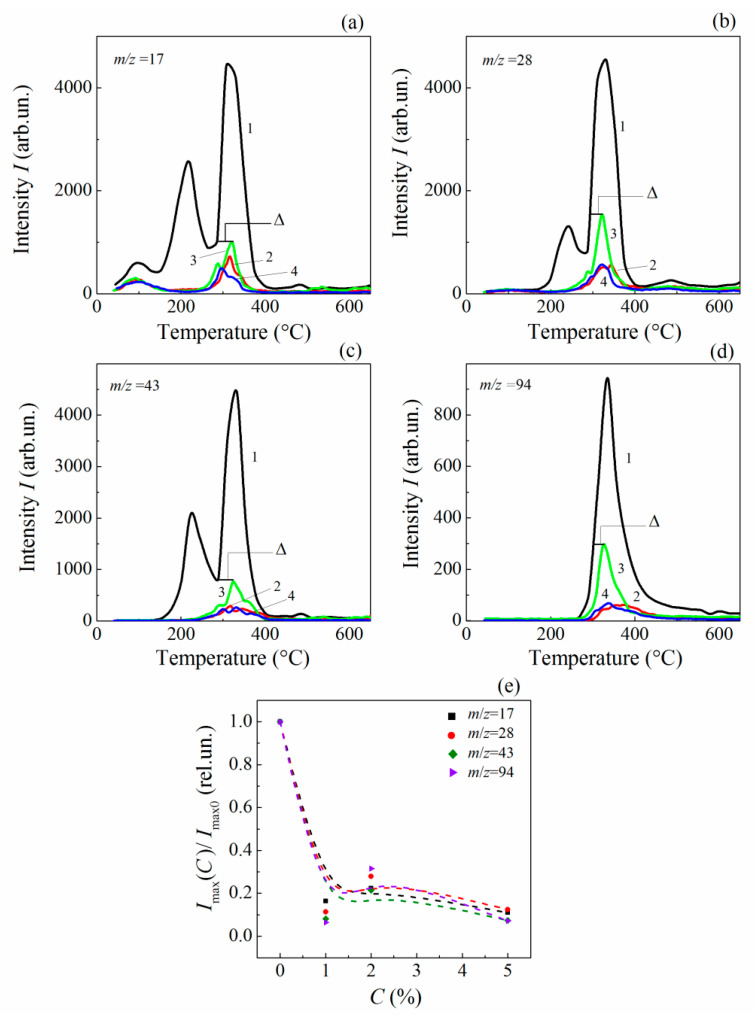
Temperature dependences of the desorption intensity of volatile fragments with *m*/*z* 17 (**a**), 28 (**b**), 43 (**c**), and 94 (**d**) for the neat epoxy (1) and MLG composites with C = 1% (2), 2% (3) and 5% (4); and related concentration dependences of normalized desorption-peak’s intensity (**e**).

Peculiarities of the MLG–epoxy interaction are clearly visible in the thermal desorption curves (Figure 7) calculated from the mass-spectra. Unbound moieties reveal themselves in the desorption curves at temperatures T ≤ 250 °C (Figure 7a,b curve 1). In the composites, desorption peaks corresponding to unbound OH- and O-containing fragments vanish (Figure 7a,b curves 2, 3 and 4). Simultaneously, the thermal destruction intensity of the built-in-network epoxy’s chains and crosslinks is significantly decreased, which occurs in the temperature range of 250–450 °C (Figure 7a,b curves 2, 3 and 4).

Normalized concentration dependences $I_{m,i}\left(C\right)/I_{m,i}\left(0\right)$ for thermal desorption peaks $I_{m,i}=I_{m,i}\left(T_{max,i}\right)$ are plotted in Figure 7e.

Thus, embedding MLGs into the host epoxy matrix significantly changes the epoxy’s molecular structure due to the interaction of ASSs with both unbound moieties and those incorporated into the macromolecular network. The decreased intensity of the thermal desorption lines indicates an increase in the thermal stability of the polymeric structure and is a consequence of the stabilization effect, which takes place in composites at low filler loading [65,67]. A mechanism for stabilizing polymer structures is the fastening of both unbound moieties and those incorporated into the macromolecular network to ASSs. It should be noted that the main contribution to the improvement in thermal stability comes from the fastened moieties incorporating the hydroxyl group and oxygen, such as OH, CO, CH_2_COH and C_6_H_5_COH (Figure 6).

It may be concluded from examination of Figure 7 that the fastening process of unbound moieties seems to be saturated at C≤ 1%. Further MLG loading has a slight effect on the desorption intensity of stabilized polymeric structure. Indeed, there is a slight nonmonotonic increase in the destruction intensity, with a maximum at C = 2% (Figure 7a,b curves 2, 3 and 4). Hence, the stabilization process is mainly fueled by the fastening of unbound moieties in the concentration range C≤ 1%, whereas fastening of network-incorporated moieties apparently dominates at C≥ 1%.

On the other hand, a nonmonotonous increase of the thermal destruction intensity with increasing C at C> 1% (see Figure 7e) may evidence a nonregular spatial alteration of polymeric structure due to a random distribution of fastening sites at the MLG–epoxy interface. Indeed, since the basal and lateral surfaces of graphene particles have a complicated morphology that is characterized by bends, pores, waviness, roughness and edge defects (see Figure 1), the ASSs locate in an arbitrary manner over the surface and thus result in a random distribution of sites of fastening. As a result, the fastening is accompanied by the formation of interphase layers around MLG particles. The interphase layers are characterized with a nonuniform distribution in both mass and bound-charge densities. Therefore, the dielectric parameters of the interface regions are essentially assumed to be increased with respect to those of the remote regions (as was discussed above, in 3.2.1).

The degree of this structural disordering is higher than that in remote regions, and it varies nonmonotonously with increasing C. It may be assumed that terminal and lateral O- and OH-containing epoxy-chain segments bearing relatively high dipole moments will “catalyze” the process of the chain’s destruction in the interphase regions. This process is capable of deteriorating the composite’s overall properties at high C values.

**Figure 8:**
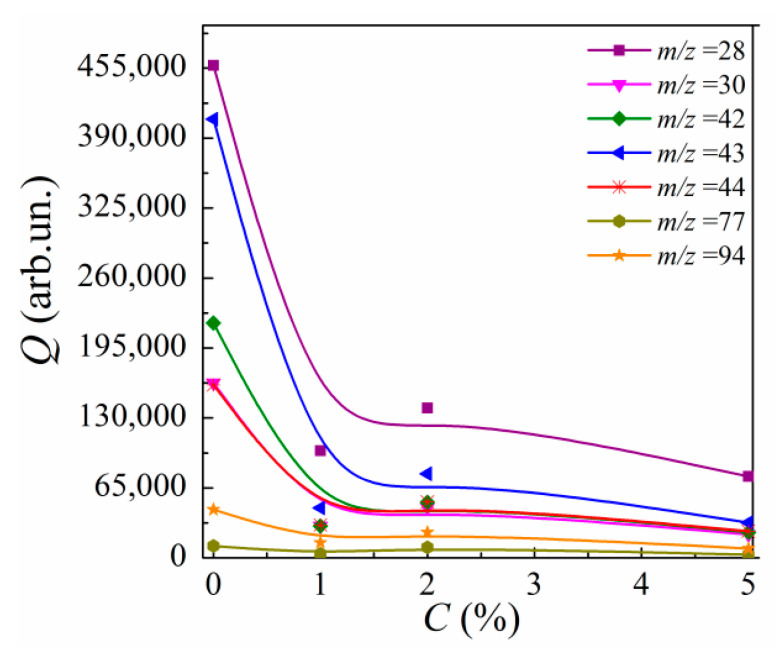
Concentration dependences of the output Q for volatile thermal desorption fragments with *m*/*z* = 28, 30, 42, 43, 44, 77, and 94 for MLG–epoxy composites.

The concentration dependences for the thermodesorbed fragment’s outputs $Q_{i}$ are shown in Figure 8. The $Q_{i}$ values were calculated using the following expression:

(1) $$Q_{i}\left(C\right)=\int_{T_{1}}^{T_{2}}I_{i}\left(T,C\right)dT$$

where $T_{1}$ = 250 °C and $T_{2}$ = 550 °C are the bounds for the temperature interval of the thermal desorption peak of intensity $I_{i}\left(T,C\right)$ for the *i*th thermally desorbed fragment of the nanocomposite of a given MLG’s concentration C. 

One can see that the $Q_{i}\left(C\right)$ curves descend steeply in the region of 0 <C< 1% and then decrease nonmonotonously, with a slight maximum at C = 2%, when C is increased. A value of $Q_{i}$ averaged over the fragments can be used as a quantitative measure of the material’s thermal stability. The lower the averaged Q value, the higher the thermal stability.

#### 3.2.2. Activation Energy of Thermal Destruction

Nonmonotonous variations in $Q_{i}$ with increasing C can be explained by the corresponding variations in the desorption activation energies $E_{d,i}$ of thermodesorbed fragments. 

The $E_{d,i}$ values were calculated by using the Polanyi–Wigner formula [68]:

(2) $$E_{d}=R\frac{T_{1,i}T_{2,i}}{T_{1,i}-T_{2},i}\ln\left(\frac{\theta_{1,i}}{\theta_{2},i}\right)$$

where R is the universal gas constant, $T_{1,i}$ − $T_{2,i}$ is the temperature width of the *i*th desorption peak width as measured at the half-height of the $I_{i}\left(T,C\right)$ curves, $\theta_{1,i}$, $\theta_{2,i}$ are the areas under the desorption curves that correspond to the amount of the ith fragment remaining after reaching $T_{1,i}$ and $T_{2,i}$, respectively. 

**Figure 9:**
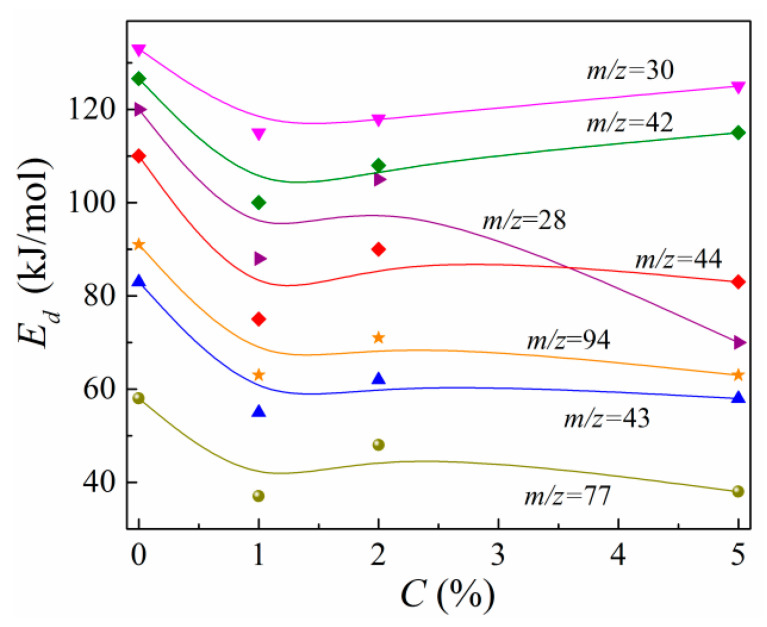
Concentration dependences of the desorption activation energy for volatile thermal desorption fragments with *m*/*z* = 28, 30, 42, 43, 44, 77, and 94.

Calculations of the activation energy for neat epoxy polymer provide $E_{d,i}$ values in the range of 55–132 kJ/mol. The $E_{d,i}$ concentration dependences are presented in Figure 9. 

It can be seen from Figure 9 that the $E_{d,i}$ values for all volatile fragments decrease noticeably at C= 1%. The $E_{d,i}$ values increase slightly and nonmonotonously with increasing C, revealing their local maxima at C= 2%, which are essentially lower than $E_{d,i}\left(0\right)$ values. It should be noted that previously, the authors of [69,70] noted the possibility for the destruction activation energy to decrease due to the incorporation of residual solvent molecules or impurities into polymer chains during the curing process. It is obvious when comparing Figure 8 and Figure 9 that the $E_{d,i}\left(C\right)$ dependences resemble those of $Q_{i}$. However, the $E_{d,i}\left(C\right)$ dependences do not correlate with the $I_{m,i}\left(C\right)$ dependences. The nonmonotonic variations in $E_{d,i}$ with C evidence the above-mentioned nonregular spatial alteration of the macromolecular structure of the epoxy in nanocomposites filled with MLGs. In other words, the random spatial interaction between ASSs of MLG nanoparticles with both unbound moieties and atoms of polymer’s chains and crosslinks leads to the formation of interphase regions with decreased destruction energy.

#### 3.2.3. Heat Transport

It is known [68] that the stabilization effect in a polymer’s molecular structure leads to an increase in polymer’s heat resistance (HR). The effect of increasing the thermal stability in the MLG–epoxy nanocomposites described above can also be regarded as increasing their HR. Besides the stabilization effect, an additional mechanism responsible for increasing the HR of MLG composites could be related to the participation of graphene’s electron subsystem in heat transport within the nanocomposite. Namely, when the various moieties are fastened on ASSs, the phonon heat flux propagating through macromolecular epoxy chains is redistributed between the phonon and electron subsystems of MLGs at the MLG–epoxy interface. As a result, the graphene’s phonon subsystem has a lower temperature at the interface than that of the epoxy’s phonon subsystem. In other words, the local decrement in the vibrational energy of surface atoms of fastened moieties takes place. This process reveals itself as a local weakening of destruction for moieties bonded with ASSs.

A phenomenological model of heat-transfer processes at the interface is presented in the following. The heat flux q entering into the unfilled polymer from an external source is transported by propagation of the phonon flux $q_{ph}^{ep}$ through the polymer chains and crosslinks into the epoxy’s bulk. Neglecting heat dissipation on the surface, defects and phonons, we can write:

(3) $$q=q_{ph}^{ep}$$

In MLG–epoxy composites, the heat is transported through chains attached to ASSs. The phonon heat flux $q_{ph}^{ep}$ is governed by the Fourier equation:

(4) $$q_{ph}^{ep}=-k_{ph}\nabla T_{ph}$$

where $k_{ph}$ is the thermal conductivity coefficient of the epoxy’s chains, $\nabla T_{ph}$ is the temperature gradient in the chains. At points of fastening, the heat is transferred from local phonons to the electronic and phonon subsystems of MLGs, and the flux obeys laws of both energy and momentum conservation. In the local approximation, we can write

(5) $$q_{ph}^{ep}=\theta \left(q_{ph}^{gr}+q_{e}^{gr}\right)$$

where θ is the heat transfer efficiency at the interface, $q_{ph}^{gr}$ and $q_{e}^{gr}$ are the phonon and electron heat fluxes, respectively. 

At high temperatures T close to chain’s destruction temperatures, the heat transfer occurs in the anharmonic mode of phonon generation, when the appearance of backscattered local phonons at the interface is unlikely and the transfer efficiency is close to unity: θ≈ 1. The heat obtained by the electronic subsystem of graphene does not return back into the epoxy’s phonon subsystem due to the absence of electron transport in the epoxy. In graphene, electron gas dissipates the received heat into the phonon subsystem due to electron–phonon interaction during the relaxation time $\tau_{r}$ at the distance s from the interface:

(6) $$s=v\tau_{r}$$

where v is the velocity of the heated electrons. In the local approximation supposed above, the interfacial temperatures of epoxy’s phonons $T_{ph}^{ep}$, graphene’s phonons $T_{ph}^{gr}$ and graphene’s electrons $T_{e}^{gr}$ obey the simple relation:

(7) $$T=T_{ph}^{ep}=T_{ph}^{gr}+T_{e}^{gr}$$

Hence, there is a temperature decrement Δ at the interphase boundary for the temperature $T_{ph}^{gr}$ as compared to $T_{ph}^{ep}$, which is given by

(8) $$\Delta T=T_{e}^{gr}=T_{ph}^{ep}-T_{ph}^{gr}$$

As a result, the vibration energy of the vibrations of the surface site’s atoms is decreased by $k_{B}\Delta T$. Therefore, the destruction probability for every *i*th moiety fixed on ASSs decreases, and its decomposition intensity $I_{i}$ also decreases.

Since different ith moieties fastened to ASSs have different masses, their vibration frequencies are different too. In addition, at the interface, the velocities $u_{i}$ and impulses $p_{i}$ of local phonons in fastened moieties are also different. Therefore, both momentum and energy values transferred from the epoxy’s phonon subsystem into the graphene electronic and phonon subsystems, as well as the temperature decrement ΔT, depend on the masses $m_{i}$ of bound atoms at the interface. The heat transfer from polymer chain to graphene takes place through local surface site.

The $\Delta T_{i}$ values can be estimated from the desorption curves of fragments with $m_{i}/z=$ 17, 43 and 94 for neat epoxy resin and its MLG composite of C= 2% in the temperature region $T<T_{m,i}$. Namely, the $\Delta T_{i}$ value is the difference between the two temperatures $T_{i0}$ and $T_{m,i}$ determined from the desorption curves $I_{i}\left(T,0\%\right)$ and $I_{i}\left(T,2\%\right)$, respectively:

(9) $$\Delta T_{i}=T_{i0}-T_{m,i}$$

Here, the temperature $T_{m,i}$ corresponds to maximal intensity $I_{m,i}$: $I_{m,i}=I_{m,i}\left(T_{m,i},2\%\right)$. When $T_{m,i}$ has been determined, one can find $T_{i0}$ from the $I_{i}\left(T,0\%\right)$ curves using the criteria: $I_{i}\left(T_{m,i},0\%\right)=I_{m,i}$. The $\Delta T_{i}$ values are shown in Figure 7. Estimations give $\Delta T_{i}\approx$ 34, 28.5, and 17 K for the fragments of $m_{i}/z=$ 17, 43, and 94, respectively (Figure 7). One can see that $\Delta T_{i}$ decreases with increasing $m_{i}$, and thus the amount of heat transferred into the graphene’s electronic subsystem decreases too.

Thus, the thermal destruction data evidence that loading epoxy resin with multilayered graphene nanoparticles results in an increase in the composite’s thermal stability in the mass-concentration range of C≤ 1%, and the thermal stability decreases slightly with increasing C. Along with the stabilization of molecular composite’s structure at C≥ 1%, the thermal destruction activation energy decreases.

### 3.3. Dielectric Parameter Data

#### 3.3.1. Dielectric Permittivity $\varepsilon_{1}$

**Figure 10:**
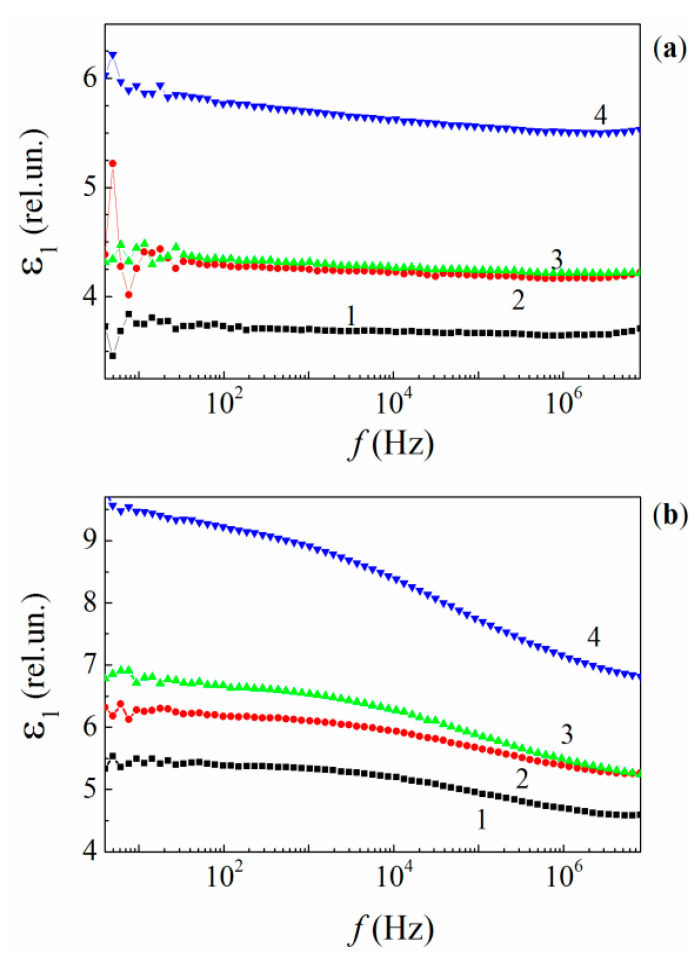
Frequency dependences of the relative dielectric permittivity for the neat epoxy (1) and MLG composites of 1% (2), 2% (3), and 5% (4) MLGs at 95 K (**a**) and 300 K (**b**).

The frequency dependences of the real part ($\varepsilon_{1}$) of the complex dielectric permittivity for the neat (unfilled) resin and its composites filled with MLG, measured at low (95 K) and room (300 K) temperature are depicted in Figure 10a,b, respectively. At low temperatures, $\varepsilon_{1}$ of the neat resin remains unchanged over the entire frequency region studied and is equal to about 3.7. Embedding MLG into the epoxy stimulates $\varepsilon_{1}$ to increase. However, the concentration dependence $\varepsilon_{1}\left(f,C\right)$ reveals a nonmonotonous variation with increasing C. Thus, $\varepsilon_{1}$ rises to 4.25–4.3 when C is increased to 1%, then it grows slowly to 4.45 when C is increased to 2%. It increases further when C is increased to 5%.

Thus, at C~ 2%, one can see a similarity in the loading behavior of the dielectric permittivity and the thermophysical characteristics, namely $Q_{i}$ and $E_{d}$. This may reveal a certain structural peculiarity of the nanocomposites.

The identical frequency behavior of $\varepsilon_{1}$ in the neat resin and its MLG nanocomposites evidence that the dielectric response is mainly caused by the epoxy’s dipolar molecular structure in all of the materials. With respect to the influence of MLG’s electron subsystem on the $\varepsilon_{1}\left(f,C\right)$ dependences, it is negligible. Hence, the concentration effect of MLG on the dielectric permittivity $\varepsilon_{1}\left(f,C\right)$ is indirect.

**Figure 11:**
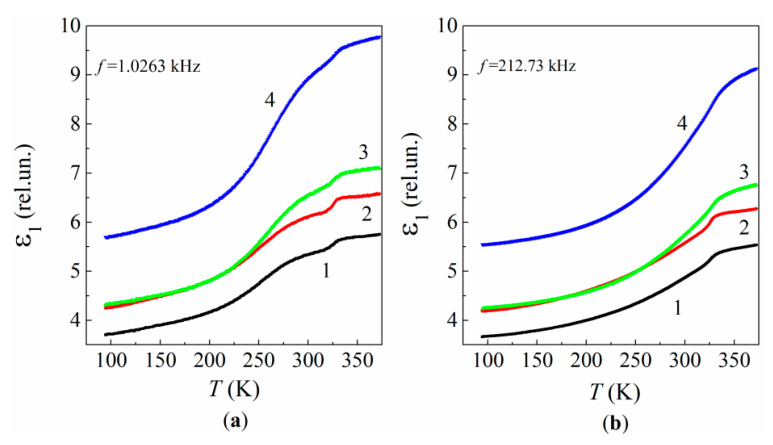
Temperature dependences of the relative dielectric permittivity for the neat epoxy (1) and MLG composites of 1% (2), 2% (3), and 5% (4) MLG at frequencies 1.0263 kHz (**a**) and 212.73 kHz (**b**).

Figure 11 shows the temperature dependences of $\varepsilon_{1}$ for the neat resin and its MLG composites measured at fixed frequencies of 1.0262 and 212.73 kHz. The dependences exhibit typical “ascending steps” pertaining to relaxation processes (see Figures 2-36b in [71]).

The concentration effect, which is clearly visible in both the frequency and temperature dependences of $\varepsilon_{1}$, seems to be related to a disordering in the polymer structure owing to the random attachment of both epoxy chains and crosslinks to the graphene’s active surface sites, which, in turn, are located randomly within the bulk epoxy. The structure disordering increases nonmonotonously with increasing C. The disordering effect prevails at C> 0.01% and leads to both increasing $\varepsilon_{1}\left(C\right)$ and decreasing $E_{d}\left(C\right)$, σ(C) and E(C).

The concentration behavior of the composite dielectric permittivity can be described by the following relation:

(10) $$\varepsilon_{1}\left(C\right)\sim \left[1-\mu \left(C\right)\right]\cdot \varepsilon_{1e}+\mu \left(C\right)\cdot \varepsilon_{1i}$$

where $\varepsilon_{1e}$ and $\varepsilon_{1i}$ are the dielectric permittivity of the initial polymer structure and the disordered structure in the interface regions formed around MLGs, respectively; μ(C) is a structural factor. The variations observed in $\varepsilon_{1}\left(C\right)$ can be explained by decreasing $\varepsilon_{1e}$, which is accompanied by increasing $\varepsilon_{1i}$ with increasing C due to structural rearrangement of polymer chains and crosslinks in the interface regions as well as the occupied interfacial volume, resulting in a more disordered and rigid structure of the polymer chains with enhanced dielectric response. However, Relation (10) fails to describe $\varepsilon_{1}\left(C\right)$ in the vicinity of 2%.

#### 3.3.2. Dielectric Losses

##### Frequency Dependences of the Dielectric Loss Factor $\varepsilon_{2}$

**Figure 12:**
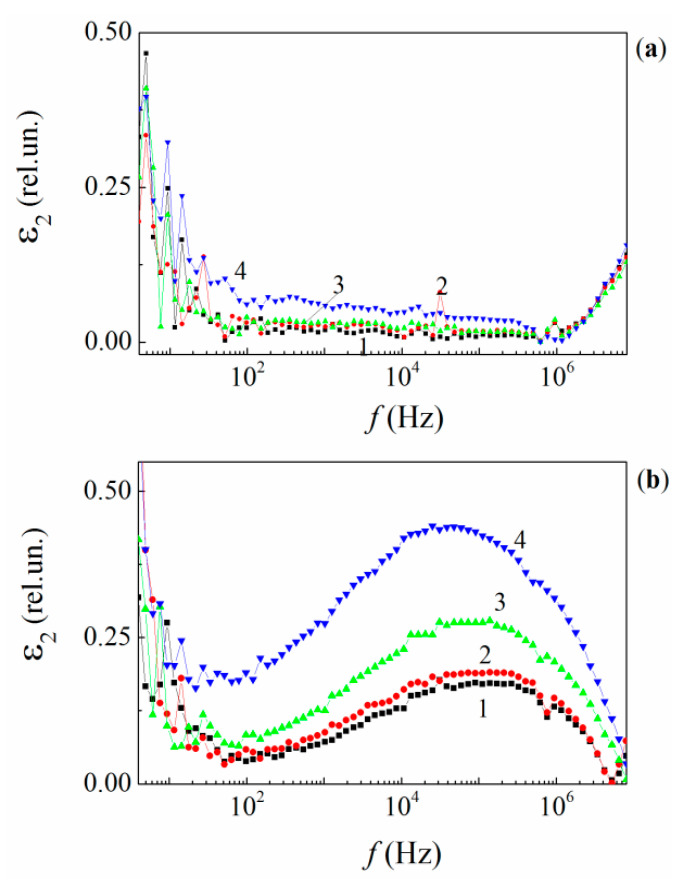
Frequency dependences of the relative dielectric loss factor for the neat epoxy (1) and MLG composites with 1% (2), 2% (3), and 5% (4) MLGs at temperatures of 95 K (**a**) and 300 K (**b**).

Frequency dependences of the dielectric loss factor $\varepsilon_{2}$ are presented in Figure 12. There are three frequency intervals where the various physical mechanisms contribute to the losses. In the narrow range of low frequencies 4–12 Hz, a sharp descending trend in $\varepsilon_{2}$ can be attributed to the term $\sigma /\omega \varepsilon_{0}$ associated with residual dc conductivity (σ) of the epoxy. On the other hand, several sharp, δ-shaped peaks are imposed on the trend. These may originate from either low mobile macromolecular atomic segments and the absorbed water molecules and/or hydroxyl groups localized on the polymer chains. However, the latter reason appears unlikely due to the fact that the loading with MLGs leads to the essential reduction in the quantity of water molecules and OH groups captured in the epoxy structure [33]. Additionally, the low-frequency losses increase with increasing C.

In the wide frequency range of 12 <f≤ 10^6^ Hz, $\varepsilon_{2}$ increases weakly with C at low temperatures. From the other hand, $\varepsilon_{2}\left(C\right)$ decreases linearly with increasing frequency up to f≈ 10^6^ Hz. It is remarkable that $\varepsilon_{2}\left(1\%\right)\approx \varepsilon_{2}\left(2\%\right)$. A further increase of $\varepsilon_{2}$ occurs when C is increased from 2% to 5%. (Figure 12b). On the whole, within the frequency interval of 12 <f≤ 10^6^ Hz, the frequency behavior of $\varepsilon_{2}$ is caused by the orientational relaxational losses of vibrating molecular dipoles of various masses and located mainly in the polymer chains due to their interaction with the dipole environment [71,72]. 

Another peculiarity of the dielectric losses is the minimum of $\varepsilon_{2}$ at f≈ 10^6^ Hz, which is followed by a steep growth with increasing frequency in the range of f> 10^6^ Hz for all the materials studied. Additionally, the $\varepsilon_{2}\left(f,C\right)$ dependences are practically identical for the unfilled resin and its MLG composites at f> 10^6^ Hz. The identical behavior of the $\varepsilon_{2}\left(f,C\right)$ curves at high frequencies suggests the mechanism for the change in the dielectric loss, namely, that the dielectric losses are related to the energy dissipation of the lateral atomic dipoles of the macromolecular chains [71,72,73].

##### Temperature Dependences of $\varepsilon_{2}$

**Figure 13:**
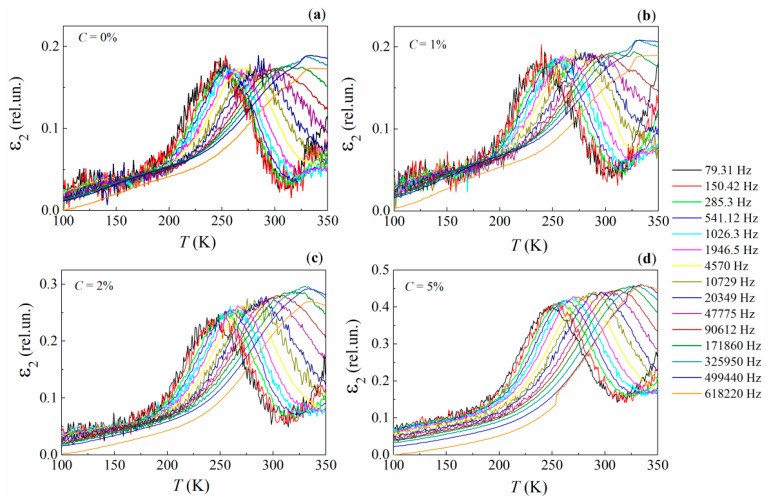
Temperature dependences of the relative dielectric loss factor (ε_2_) at fixed frequencies for the neat epoxy (**a**) and its MLG composites of 1% (**b**), 2% (**c**), and 5% (**d**) graphene particles. Fixed frequencies (in Hz) are 79.31, 150.42, 285.3, 541.12, 1026.3, 1946.5, 4570.0, 10,729.0, 20,349.0, 47,775.0, 90,612.0, 171,860.0, 325,950.0, 449,440.0, and 618,220.0.

As temperature increases, several broad relaxation peaks become visible in the temperature dependences of $\varepsilon_{2}$ measured at fixed frequencies for both the neat resin and their nanocomposites. The sets of $\varepsilon_{2}\left(T,C\right)$ curves measured at fixed frequencies are depicted in Figure 13. It is generally accepted that the relaxation mechanism responsible for the appearance of these peaks is that referred to as “the dipole-group relaxation”, which originates mainly from local vibration modes of various ensembles of dipolar moieties [71].

At low temperatures −130 ≤T≤ 50 K, several $\varepsilon_{2}\left(T\right)$ curves contain two slight peaks within the frequency range of f≤ 500 Hz (Figure 13a,b). Both the peaks can be ascribed to γ relaxators [52,74,75]. Both the peaks decrease and narrow with increasing C and become invisible at C = 5% (see Figure 13d).

The γ peaks can be attributed to low mobile unreacted molecular fragments of polymer structure and long dipole segments in the polymer chains [74,75]. The suppression of γ peaks with increasing C is caused by the decreased free volume reduction in the matrix and the fastening of chain segments on the active surface sites of the MLGs.

The most intensive relaxation losses in the neat epoxy and its MLG composites occurs at higher temperatures T> 220 K, within the frequency range 12–10^6^ Hz. These $\varepsilon_{2}$ peaks are caused by the β relaxation process, which is fed by various dipolar moieties [74,75]. The temperature position of the peaks ($T_{\beta }$) shifts toward higher temperatures when measuring frequency increases (see Figure 12), as predicted by the classical Debye theory [71].

**Figure 14:**
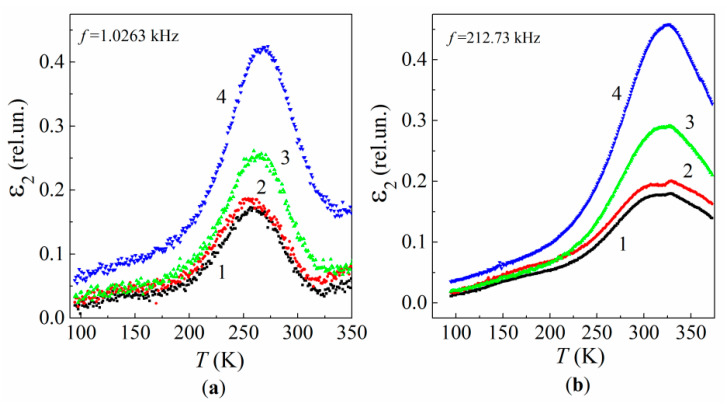
Temperature dependences of the relative dielectric loss factor at frequencies 1.0263 kHz (**a**) and 212.73 kHz (**b**) for the neat epoxy (1) and MLG composites of 1% (2), 2% (3), and 5% (4) MLGs.

It is remarkable that the dependence of $T_{\beta }$ versus C varies with increasing frequency (Figure 14). For frequencies f< 2000 Hz, $T_{\beta }$ decreases when C< 1%, and increases when C> 1% (Figure 14a). In contrast, for frequencies f> 2000 Hz, $T_{\beta }$ increases over the entire concentration interval (Figure 14b).

The nonmonotonous $T_{\beta }\left(C\right)$ dependence in the frequency range of f≤ 2000 Hz and with low MLG content C≤ 1% is caused by two competing processes. The first process occurs at low MLG content C≤ 0.01% and consists of the spatial fixing of low-movable unbound segments of polymer chains. This process leads to increased free volume of the polymer structure and spatial ordering. Such structural transformation manifests itself in slight variations in the losses and in a low-temperature $T_{\beta }$ shift. The second process dominates at 0.01 <C≤ 1% and consists of the fastening of polymer chains and crosslinks on the active surface sites of the MGLs and the disordering of the polymer structure due to the formation of disordered interface regions. Competition of the two processes results in a decrease in $T_{\beta }$ along with a slight enhancement of dielectric losses (Figure 14a, curve 2).

Thus, the presence of unbound fragments of polymer chains and their attachment to the active surface sites of MLG particles, as well as the change of MLG content in the polymer matrix are the key factors affecting the degree of disorder in the polymer structure and the energy distribution of molecular dipoles.

Using a set of $\varepsilon_{2}\left(T,C\right)$ temperature dependences measured at different frequencies, the activation energy $E_{\beta }$ of dipole relaxation can be determined as a function of C. In accordance with the classic Debye theory [71], at the relaxation resonance temperature $T_{\beta }=T_{\beta }\left(C\right)$ the dipole resonance frequency $\omega_{\beta }=\omega_{\beta }\left(C\right)$ is related to relaxation time ($\tau_{\beta }$) by the following expression [71]:

(11) $$\omega_{\beta }\left(C\right)\cdot \tau \left(T_{\beta }\right)=1$$

where the τ value depends on the Boltzmann probability of the energy distribution of vibrating dipoles. The probability term is taken into account as follows:

(12) $$\tau \left(T_{\beta }\right)=\tau_{\beta 0}\left(C\right)exp\left[E_{\beta }\left(C\right)/k_{B}\cdot T_{\beta }\left(C\right)\right]$$

where $\tau_{\beta 0}$ is the frequency-independent time parameter of the β relaxation process, $k_{B}$ is the Boltzmann constant. The formula to determine $E_{\beta }\left(C\right)$ is

(13) $$ln\left(\frac{1}{\omega_{\beta }\left(C\right)}\right)=ln\left[\tau_{\beta }\left(C\right)\right]+\frac{E_{\beta }\left(C\right)}{k_{B}\cdot T_{\beta }\left(C\right)}$$

**Figure 15:**
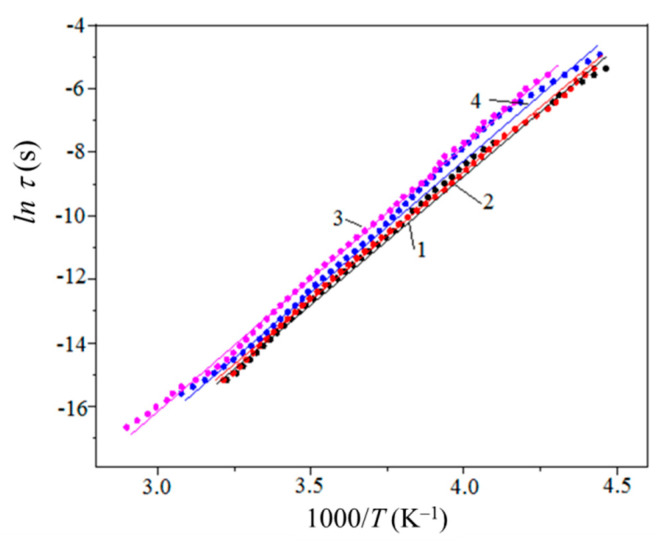
Temperature dependences of the logarithm of the dipole relaxation time for the neat resin (1) and MLG composites of 1% (2), 2% (3), and 5% (4) MLGs.

The set of temperature dependences of $ln\left[\tau \left(T_{\beta }\right)\right]$ show that the relaxation process exhibits about the same activation energy for both the neat resin and its MLG composites (Figure 15). Namely, $E_{\beta }\left(0\%\right)\approx E_{\beta }\left(1\%\right)$ = 0.70 eV and $E_{\beta }\left(2\%\right)\approx E_{\beta }\left(5\%\right)$ = 0.72 eV. This means that the atomic environment for the epoxy’s β dipoles undergoes a negligible variation at low loadings C≤ 2%.

### 3.4. Positron Annihilation Lifetime Data

Lifetime positron annihilation spectroscopy was applied to probe molecular structure alterations in the MLG–epoxy nanocomposites. Three positron lifetimes ($\tau_{n}$) and corresponding annihilation intensities ($I_{n}$) were determined. $(\tau_{1}$, $I_{1})$, $(\tau_{2}$, $I_{2})$ and $(\tau_{3}$, $I_{3})$ describe the annihilation of the quasi-free positrons, the trapped positrons, and the trapped positronium atoms, respectively, while the same experimental annihilation parameters ($\tau_{n}$, $I_{n}$) and the calculated ones (namely, the average positron lifetime $\tau_{av}$, the radius of positronium-capturing traps $R_{Ps}$, and the free volume $f_{v}$) are presented in Table 1.

Here, $\tau_{av}$ was calculated on the basis of the following expression [76]:

(14) $$\tau_{av}=\left(I_{1}\tau_{1}+I_{2}\tau_{2}+I_{3}\tau_{4}\right)/\left(I_{1}+I_{2}+I_{3}\right)$$

**Figure 16:**
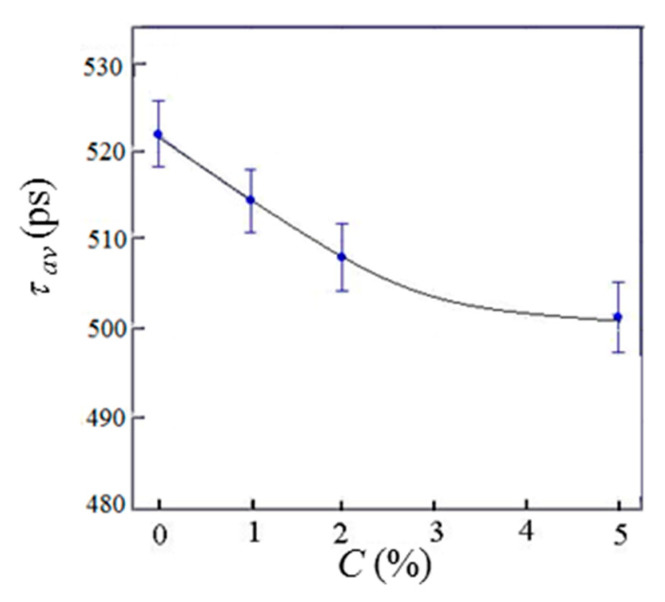
Concentration dependence of the average positron lifetime for MLG–epoxy composites.

$\tau_{av}$ decreases smoothly with increasing C (see Figure 16). Since $\tau_{av}$ is inversely proportional to the electron density $\left(n_{e}\right)$ in the annihilation volume, lowering $\tau_{av}$ means increasing $n_{e}$.

Despite increasing $n_{e}$, both $I_{1}$ and $\tau_{1}$ take their minimal values at C= 2%. It is remarkable that $\tau_{1}\left(C\right)$ dependence is essentially nonmonotonous. Indeed, $\Delta \tau_{11}\left(C\right)=\tau_{1}\left(0\%\right)-\tau_{1}\left(1\%\right)=$ 0.2 ps, but $\Delta \tau_{12}\left(C\right)=\tau_{1}\left(1\%\right)-\tau_{1}\left(2\%\right)=$ 8.7 ps. The low value of $\Delta \tau_{1}$ may be attributed to the influence of two opposite processes of structural alteration. The first process fastens the unbound moieties on ASSs. This leads to decreased disorder in the macromolecular structure, and is accompanied by increased $\tau_{1}$, and thus by a decrease in electron density in the positron’s annihilation volume. Another process is the formation of interphase layers around MLGs due to the nonuniform spatial distribution of ASSs, resulting in enhanced disorder within the polymer structure due to the random process of the chemical binding of atoms in polymer chains and crosslinks with the surface sites of graphene particles. Superposition of the two processes results in decreasing $\tau_{1}$ and a corresponding increase in the electron density along epoxy chains and crosslinks in the annihilation volume.

The structural variations at C= 2% also manifest themselves in local maxima of $I_{2}$ and $I_{3}$. They evidence an increasing number of nanosized pores with radii of about 2.4–2.5 Å, since $I_{2}$ and $I_{3}$ are proportional to the densities of the corresponding pores. Again, the local minima of $\tau_{2}$ and $\tau_{3}$ at C= 2% can be attributed to the increasing electron density on the walls of the corresponding pores trapping positrons and ortho-positronium atoms, respectively.

**Figure 17:**
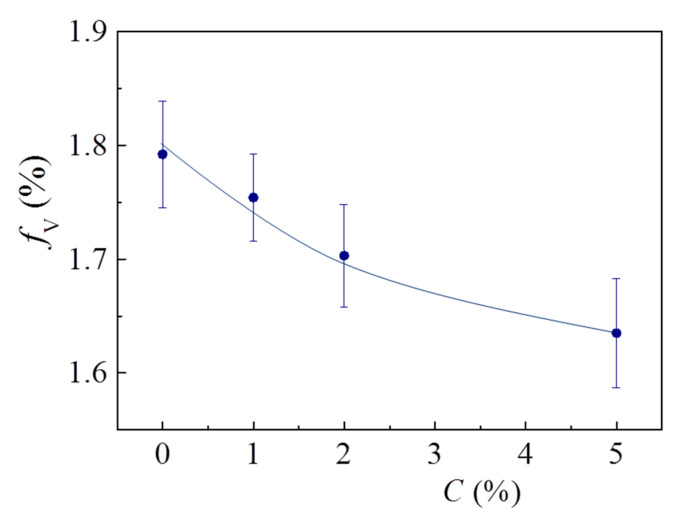
Concentration dependence of the free volume for MLG–epoxy composites.

The concentration dependence of free volume $f_{v}$ is presented in Figure 17. $f_{v}$ values were calculated using the following well-known formula [77]:

(15) $$f_{v}=0.018\cdot I_{3}\cdot \left(\frac{4}{3}\pi R_{PS}^{3}\right)$$

where $R_{Ps}$ is the radius of positronium-trapping pores (voids), evaluated by solving the following equation [78]:

(16) $$\tau_{3}^{-1}=\frac{1}{2}\left[1-\frac{R_{Ps}}{R_{0}}+\frac{1}{2\pi }\sin\left(\frac{2\pi R_{Ps}}{R_{0}}\right)\right]$$

where $R_{0}=R_{Ps}+\Delta R$ and ΔR = 1.66 Å is the fitting parameter [78].

The free volume was found to decrease with increasing C and tend to saturation at C> 5% (Figure 17). Such behavior is correlated with a smooth growth of the mean electron densities in polymer chains and on the walls of positron- and positronium-trapping pores. It is remarkable that $\tau_{av}\left(C\right)$ and $f_{v}\left(C\right)$ resemble each other. This confirms the conclusion that MLG filler compacts the macromolecular structure of epoxy smoothly, and the mean electron density (which is proportional to ${\left(\tau_{av}\right)}^{-1}$ in the annihilation volume increases as the free volume portion decreases.

However, the structure’s constituents, such as epoxy chains, crosslinks, and nanosized defects (such as pores), are characterized by nonmonotonous alteration with MLG loading. In the concentration range 0 <C≤ 1%, when unbound moieties fasten to ASSs and the macromolecular network structure undergoes slight conformational deformation, PALS-data reveal a weak increase in electron density in the epoxy chains (which is proportional to ${\left(\tau_{1}\right)}^{-1}$), along with an increasing number of positronium-trapping pores (which is proportional to $I_{3}$).

In the concentration range 1 <C≤ 2%, both the free volume ($f_{v}$) and the conformational deformation (Δε) decrease, and the chain’s electron density ($\sim {\left(\tau_{1}\right)}^{-1}$) decreases too. In addition, the numbers of both positron-trapping pores and positronium trapping pores increase due to increasing $I_{2}$ and $I_{3}$, respectively (see Table 1). The electron densities on the pore walls increase due to decreasing $\tau_{2}$ and $\tau_{3}$, respectively. It can be easily seen from Figure 10a that $\varepsilon_{1}\left(f\right)$ undergoes negligible variations in the range of 1 <C≤ 2%. This behavior in $\varepsilon_{1}$ is caused by the effect of “breeding” the pores.

In the concentration range 2 <C≤ 5%, the numbers of both positron-trapping pores and positronium-trapping pores decrease and the electron densities on the pore walls decrease too (see Table 1). The dielectric permittivity begins to increase again, along with a further decrease in the conformational deformation.

Thus, the peculiarity observed in the macromolecular structure of the 2%-nanocomposite, which is related to the intensive formation of nanopores, reveals itself in overall physical characteristics such as thermal stability, destruction activation energy, and dielectric permittivity.

## 4. Conclusions

The effect of low concentration loading of multilayered graphene particles on thermal stability, compressive strength and Young’s modulus, dielectric permittivity, dielectric losses, and the free volume of the epoxy composites were studied.

At C≤ 5%, the MLG loading leads to an essential increase in thermal stability and a decrease in destruction activation energy.

The compressive strength and the Young’s modulus are increased at extremely low loadings of C≤ 1% and decrease when C is increased to 1%.

At C≤ 1%, fastening unbound moieties on ASSs stabilize the composite’s molecular structure, resulting in the enhanced thermal stability of the composite. The enhancement of composite’s thermal stability may occur due to a partial transfer of heat from polymer chains fastened on graphene’s active surface sites into the electron subsystem of the MLGs.

At C> 1%, the fastening process is accompanied by the formation of interphase areas around the MLG particles, which may be due to the random distribution of fastening sites across the MLG–epoxy interface. In turn, the formation of interphase areas leads to an increase in the dielectric permittivity of the composites.

Structural alteration in the 2% nanocomposite is related to a spontaneous increase in the density of nanovoids, which enhances the dielectric response of the nanocomposites to the external electric field.

At 0% <C≤ 5%, both the free volume and the mean electron density in the composite’s bulk decrease gradually with increasing C.

## Figures and Tables

**Table 1:** Positron annihilation parameters for epoxy and its composites with 1, 2, and 5% multilayer graphene nanoparticles.

C,%	$I_{1},\%$	$I_{2},\%$	$I_{3},\%$	$\tau_{1},\;\mathrm{ps}$	$\tau_{2},\;\mathrm{ps}$	$\tau_{3},\;\mathrm{ps}$	$\tau_{av},\;\mathrm{ps}$	$R_{Ps},\;Å$
0	53.23	31.96	14.82	199. 6	527.0	1670	522.1	2.522
1	52.86	31.82	15.32	199.4	503.0	1627	514.6	2.476
2	49.66	34.52	15.82	190.7	473.5	1581	508.3	2.426
5	52.68	32.45	14.88	199.9	489.0	1597	501.6	2.443

## Data Availability

Not applicable.
